# Potential compensatory mechanism for cognitive impairment in type 2 diabetes and prediabetes: altered structure-function coupling

**DOI:** 10.3389/fendo.2025.1491377

**Published:** 2025-03-17

**Authors:** Weiye Lu, Xuan Huang, Die Shen, Kun Wang, Jiahe Wang, Ziyu Diao, Shijun Qiu

**Affiliations:** ^1^ The First Clinical Medical College, Guangzhou University of Chinese Medicine, Guangzhou, Guangdong, China; ^2^ Department of Radiology, The First Affiliated Hospital of Guangzhou University of Chinese Medicine, Guangzhou, Guangdong, China; ^3^ State Key Laboratory of Traditional Chinese Medicine Syndrome, Guangzhou, Guangdong, China

**Keywords:** type 2 diabetes mellitus, diffusion-weighted MRI, resting state functional MRI, structure-function coupling, cognitive impairment

## Abstract

**Background:**

Structure-function (SC-FC) coupling may be more sensitive to detecting changes in the brain than any single modality. The aim of this study was to investigate the effects of SC-FC coupling changes on cognition and their interactions in patients with prediabetes and type 2 diabetes mellitus (T2DM).

**Methods:**

A total of 493 participants (119 with normal glucose metabolism (NGM), 125 with prediabetes, and 249 with T2DM) were included in the study. Diffusion-weighted MRI and resting state functional MRI data were used to quantify SC-FC coupling. General linear model and linear regression analysis were used to evaluate the relationship between glucose metabolism, SC-FC coupling, and cognition. Mediation models were used to evaluate the mediating role of regional SC-FC coupling between diabetes-related measures and cognition.

**Results:**

The regional coupling strength of SC-FC varied greatly in different brain regions, but was strongest in the ventral attention and somatmotor network areas. Compared with NGM patients, T2DM patients had higher SC-FC coupling in the default mode network but lower SC-FC coupling in the limbic network. In addition, fasting glucose and HbA_1c_ were associated with weaker SC-FC coupling in the limbic network, fasting insulin with higher SC-FC coupling in the limbic network, and HbA_1c_ with higher SC-FC coupling in the dorsal attention network. Furthermore, through mediated models we found that SC-FC coupling in the limbic network suppressed the association between diabetes-related measures and cognition.

**Conclusion:**

T2DM and diabetes-related measures were associated with abnormal SC-FC coupling of the limbic network. The recombination of SC-FC coupling relationships in the limbic network may indicate a potential compensatory mechanism for cognitive decline that begins in prediabetes.

## 1 Introduction

The global prevalence of diabetes will continue to rise in the future due to population aging, with increases predicted to be more noticeable in low- and middle-income countries (1). Cognitive impairment is widely recognized as a prevalent comorbidity of type 2 diabetes mellitus (T2DM), with minor cognitive alterations observed in individuals across all age categories, but more severe stages are typically observed in older adults aged 65 years and order (2). The exact underlying causative mechanism of cognitive decline in T2DM patients has not been fully identified and is almost certainly complex, involving multiple interacting variables (3). Hyperglycemia, obesity, and insulin resistance can induce brain microvascular dysfunction, which is already evident in adults with prediabetes (4–7), leading to brain structural abnormalities such as vascularized lacunae, cerebral microbleeds, perivascular spaces, global brain atrophy, and microinfarcts (8), and there is growing evidence that this is one of the key drivers of cognitive decline (9). Alterations in neural connections could be a key step in microvascular dysfunction that results in cognitive impairment (10). An ongoing population prospective cohort study found that functional connections between the right thalamus and the visual network were lower in T2DM patients and was associated with cognition (11). The Maastricht Study cohort reported reduced white matter connections between the hippocampus and frontal lobes in T2DM patients and was associated with memory impairment (12). Despite these discoveries, the link between cognitive impairment and abnormal brain structural and functional connectivity remains controversial. Alterations in structural and functional connectivity may contribute to cognitive dysfunction, but they may also represent compensatory and protective mechanisms. Therefore, a single-modal evaluation of brain connectivity is inadequate to fully comprehend the underlying brain mechanisms of T2DM-related cognition. Structure-function (SC-FC) coupling quantifies the correlation between structural and functional connectivity, indicating consistency across networks, which can serve as a marker to detect subtle pathological abnormalities (13, 14). In addition, SC-FC coupling is more sensitive in detecting brain abnormalities than a single model (14), possibly due to the fact that multimodal data integration can reduce noise or bias from a single mode and improve the robustness of the results. The existing literature lacks a joint analysis of multimodal MRI for diabetic status, and the relationship between structural and functional connections remains to be clarified.

Our study performed the first comprehensive analysis of cerebral abnormalities combined with structural and functional connections in individuals with prediabetes and T2DM. We aimed to investigate differences in populations with different glucose metabolism statuses by measuring SC-FC coupling levels and their characteristic relationship with cognitive function. We hypothesize that abnormalities in SC-FC coupling similar to T2DM are already present in prediabetes to a lesser extent, and that this abnormality mediates or compensates for the decline in cognitive function.

## 2 Materials and methods

### 2.1 Participants

A cross-sectional and observational study was done on 600 participants who were recruited from outpatient and inpatient diabetes clinics at the Guangzhou University of Traditional Chinese Medicine between 2020 and 2023. The inclusion criteria were right-handedness and age 30-70 years. 566 of the 600 participants had MRI scans available. Among the 566 participants with accessible MRI measures, 493 with complete clinical data and images (**Supplementary Figure S1**). Committee approval (NO. K-2013-146) was obtained from Guangzhou University of Traditional Chinese Medicine’s First Affiliated Hospital for the study. All were asked to sign a written informed consent prior to participating in the study.

### 2.2 Prediabetes and T2DM status

Patients with a history of diabetes mellitus were tested for fasting plasma glucose (FPG), a standardized 2-h 75-g oral glucose tolerance test, and HbA_1c_ levels. Participants without a history of diabetes were tested for FPG and HbA_1c_ levels. All trials were completed at the First Hospital of Guangzhou University of Traditional Chinese Medicine. The classification of prediabetes and T2DM was determined based on the diagnostic criteria provided by the American Diabetes Association in 2021 (15). Participants were classified as having prediabetes if their FPG was between 5.6~6.9 mmol/L and/or HbA_1c_ was between 5.7~6.4%. The diagnosis of T2DM was confirmed by a self-reported history of diabetes, use of hypoglycemic drugs, FPG ≥7.0 mmol/L, 2-h postload glucose ≥11.1 mmol/L, or HbA_1c_ ≥6.5%. Normal glucose metabolism (NGM) is defined as FPG and HbA_1c_ within the normal range.

### 2.3 MRI and image preprocessing

The 64-channel 3.0T MRI scanner (Prisma, Siemens, Germany) was used to obtain 3D T1-weighted imaging, diffusion-weighted MRI (dMRI) and resting-state functional MRI (fMRI), and the scanning parameters are shown in **Supplementary Table S1**. All neuroimaging data underwent a thorough visual examination to rule out artifacts and inaccurate segmentation and registration. If the head movement was more than 3 mm in three dimensions or 3 degrees in rotation, images were not included. The preprocessing of fMRI data was using SPM 12 software. The processing steps included removing the first 10 time points, correcting for slice timing and head motion, normalizing to Montreal Neurological Institute coordinates using the DARTEL method, applying spatial smoothing with Gaussian kernels of 6 mm full width at half maximum (FWHM), linear detrending, and regressing global signal, cerebrospinal fluid signals, white matter, and Friston-24 head motion parameters. Ultimately, the impacts of high-frequency physiological noise and low-frequency drift were mitigated by using a bandpass filter with a frequency range of 0.01 to 0.08 Hz.

The DTI data underwent preprocessing and analysis using the PANDA v1.3.1 toolbox (http://www.nitrc.org/projects/panda). Rigid-body transforms were performed on the b0 images to correct eddy current distortions and head motion artefacts. The Diffusion Toolkit was used to estimate diffusion tensor models at each voxel using the linear least-squares fitting approach. The Diffusion Toolkit’s Fiber Assignment by Continuous Tracking technique was applied to track whole brain fibers in native diffusion space. Streamlines tracking was stopped when the angle between the current and the previous path segment surpassed 45° or the FA was less than 0.2.

### 2.4 Construction of functional connectivity

In order to measure the strength of functional connectivity (FC), we calculated the Pearson correlation between the average time series of each pair of brain areas, utilizing the Human Brainnetome Atlas (16). As a result, each participant has a 246×246 FC matrix with a Gaussian distribution after Fisher’s Z transformation. The components of negative correlations were set to zero due to the contentious physiological interpretation of negative correlations (17, 18).

### 2.5 Construction of structural connectivity

In order to obtain structural connectivity (SC), we defined each region of interest (ROI) as a network node in the Human Brainnetome Atlas. The Human Brainnetome Atlas from the MNI space was registered to the individual’s native space via inverse transformations. In native diffusion space, structural connectivity was considered to be present if streamlines were present in at least 80% of participants in each pair of brain regions. We calculated the weight of each edge by taking the mean FA values of all the fibers.

**Figure 1:**
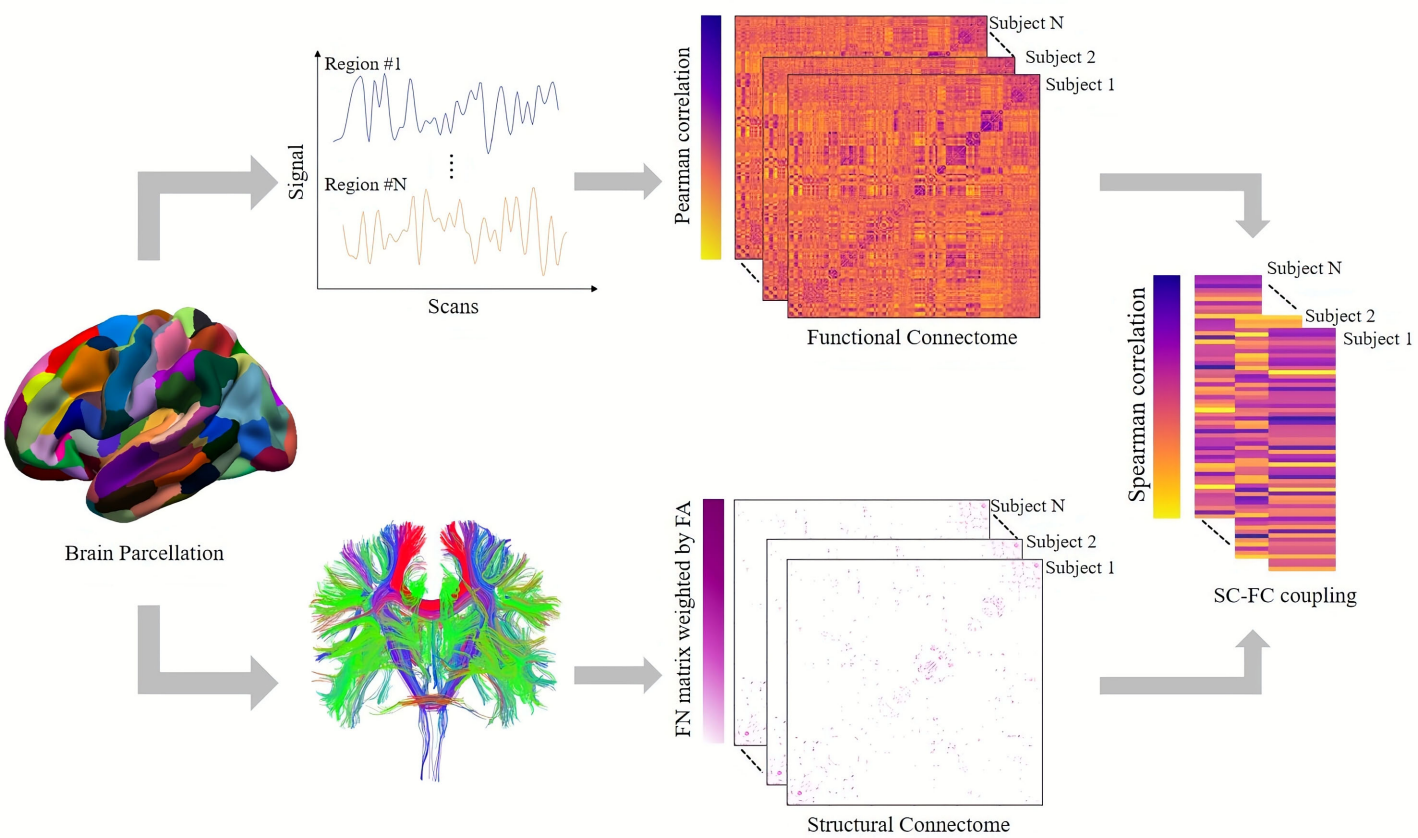
Presentation of the SC-FC coupling process. The Human Brainnetome Atlas divides the cortical and subcortical brain regions into 246 regions of interest. SC matrix reconstructs white matter fibers by deterministic tracing algorithm. The FC matrix was constructed by measuring the Pearson correlation between the mean time series of each pair of brain regions. The SC-FC coupling value was obtained by measuring the Spearman correlation coefficient between non-zero SC and corresponding FC.

In order to analyze the alterations in the SC-FC coupling at the network level, we implemented the classic seven-network parcellation according to Yeo atlas (19), which includes the visual network (VIS), somatomotor network (SOM), ventral attention network (VEN), dorsal attention network (DOR), limbic network (LIM), frontoparietal network (FPN), and default mode network (DMN). Subcortical regions were included in the eighth network. The procedure for constructing the network is outlined in **Figure 1**.

### 2.6 SC-FC coupling analysis

Correlation coefficients between structural and functional connectivity are used to measure SC-FC coupling. This correlation was limited for each participant by the edges of non-zero structural connection. To be more precise, a vector of structural connectivity values was formed by extracting non-zero structural connectivity edges, and a corresponding vector was created by extracting functional connectivity. The SC-FC coupling values were then obtained by measuring the correlation coefficient between the two priori vectors using Spearman’s correlation. Lastly, tractography algorithms are known to underestimate connectivity across different hemispheres. Consequently, we also compute the SC-FC coupling inside a hemisphere.

### 2.7 Statistical analysis

The characteristics of the participants were expressed as mean ± SD (continuous variables) and percentage (categorical variables). Comparisons between glucose metabolism status were conducted using statistical analyses appropriate for the type of data. ANOVA was used for continuous variables that followed a normal distribution; the Kruskal-Wallis test was used for continuous variables that exhibited a skewed distribution; and the categorical variables were analyzed using the Pearson χ^2^ test. A *post hoc* test was then performed, using two-sample t tests for means and χ^2^ test for proportions. If the SC-FC coupling values conform to the normal distribution, a general linear model (GLM) is used for statistical analysis; otherwise, a non-parametric permutation test is used, followed by a *post hoc* test (*P* <.05). The analyses were controlled for potential confounding variables, specifically age, sex, and education level (model 1). Additional adjustments were made for cardiovascular disease risk factors, including BMI, office systolic blood pressure, the ratio of total cholesterol to HDL, lipid-modifying medication, antihypertensive medication, and history of cardiovascular disease (model 2). The variance inflation factor was used to test for multicollinearity of the independent variables. Interaction terms for sex * glucose metabolism status were added to explore the interaction. The analyses were conducted using SPSS (IBM, SPSS, version 27).

To investigate the association between diabetes-related measures, regional SC-FC coupling, and the Montreal Cognitive Assessment (MoCA) score (20), and to explore whether the brain SC-FC coupling acts as a potential mediator in the association between diabetes-related measures and MoCA score, we adopted a two-stage statistical analysis. In the first stage, multiple linear regression analysis was used to examine the relationship between glucose metabolism status, diabetes-related measures, and regional SC-FC coupling. Dummy variables were then used to evaluate regression coefficients for different glucose metabolism groups. The correction for confounding variables is consistent with the above. Secondly, based on the significant association between exposure (diabetes-related measures) and mediator (regional SC-FC coupling), as well as the association between the mediator and the outcome (MoCA score), we performed the mediation analysis to examine whether brain SC-FC coupling metrics mediated the association between exposure and outcome. To calculate bias-corrected 95% CIs, we used bootstrapping (5000 samples) and the PROCESS statistical package for SPSS (21).

## 3 Results

### 3.1 Population characteristics

**Table 1** displays the baseline demographic information. Out of 493 people who were part of the study, 119 were NGM, 125 were prediabetes, and 249 were T2DM. The average age was 50.6 years, and 50.1% were female. Participants with prediabetes and T2DM were significantly older and had higher cardiovascular risks than NGM individuals (**Table 1**).

**Table 1:** Characteristics of the population.

	NGM	Prediabetes	T2DM	*P*
Demographics				
Age (years)	47.0 ± 7.0	52.9 ± 8.5	51.1 ± 8.6	<0.001
Female sex (%)	59.7	64.8	38.2	<0.001
Education (years)	10.4 ± 5.0	9.1 ± 4.2	10.9 ± 4.1	<0.001
Glucose metabolism				
Fasting glucose (mmol/L)	4.9 ± 0.4	5.3 ± 0.5	9.3 ± 3.0	<0.001
HbA_1c_ (%)	5.5 ± 0.3	5.9 ± 0.2	8.7 ± 2.3	<0.001
Diabetes duration (years)	6.0 ± 5.8	–	–	–
Fasting insulin (mIU/mL)	8.8 ± 4.3	11.6 ± 11.1	10.1 ± 8.9	0.033
Cardiovascular risk factors				
BMI (kg/m^2^)	23.3 ± 3.5	24.1 ± 3.1	24.2 ± 3.2	0.003
Systolic BP (mmHg)	118.2 ± 14.8	128.8 ± 18.9	127.8 ± 16.1	<0.001
Diastolic BP (mmHg)	77.9 ± 10.2	83.8 ± 11.6	84.2 ± 10.9	<0.001
Hypertension (%)	5.0	24.0	29.3	<0.001
Ratio of total to HDL cholesterol	3.6 ± 1.2	3.9 ± 1.1	4.6 ± 1.5	<0.001
Triglycerides (mmol/L)	1.3 ± 1.4	1.5 ± 0.9	2.3 ± 2.3	<0.001
History of cardiovascular disease (%)	4.2	9.6	12.4	0.044
Medication use				
Insulin use (%)	46.2	–	–	–
Antihypertensive medication (%)	9.2	23.2	37.8	<0.001
Lipid-modifying medication (%)	7.6	15.2	40.6	<0.001
MoCA	25.5 ± 3.6	25.2 ± 4.3	25.1 ± 3.1	0.653
Lifestyle factors				
Alcohol consumption (%)	15.1	16.0	22.9	0.118
Smoking status (%), never/former/current	67.2/22.7/10.1	69.6/21.6/8.8	63.5/20.5/16.0	0.284

Data are presented as mean ± SD or percentage. ANOVA and χ^2^ tests compare continuous and categorical variables, respectively. BP, blood pressure; MoCA, Montreal Cognitive Assessment.

### 3.2 Spatial variation of SC-FC coupling

**Figure 2:**
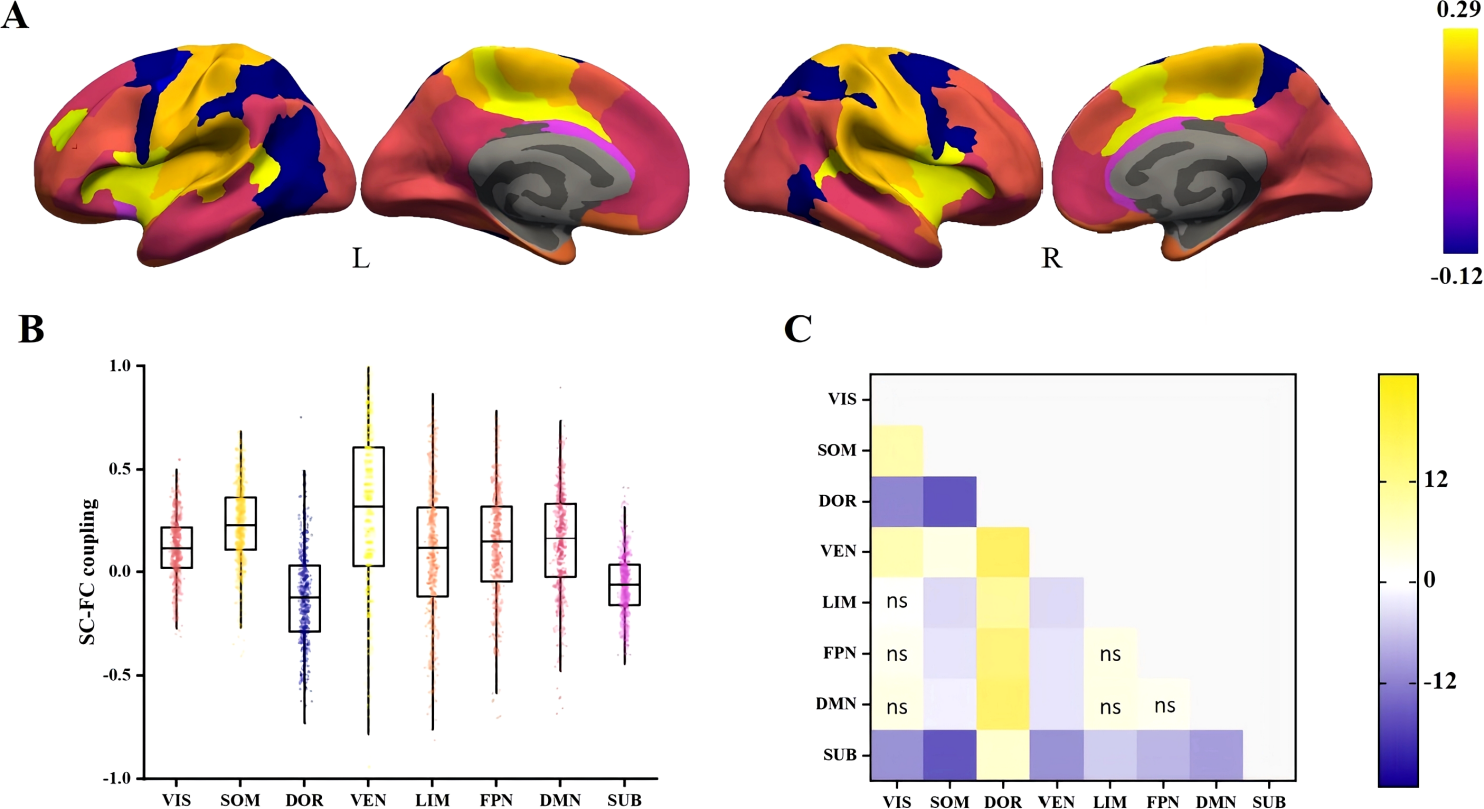
Spatial differences in SC-FC coupling. **(A)** SC-FC coupling in cortical and subcortical regions of the study population. **(B)** Distribution of SC-FC coupling in eight different networks. **(C)** The t-statistics for pairwise comparisons of SC-FC coupling between networks, calculated as the y-axis vs the x-axis. FDR-corrected comparisons with *p* > 0.05 were represented by ns. VIS, visual; SOM, somatomotor; DOR, dorsal attention; VEM, ventral attention; LIM, limbic; FPN, frontoparietal; DMN, default mode; SUB, subcortical.

**Figure 2** illustrates the average SC-FC coupling among 493 unrelated individuals. Regional SC-FC coupling varied substantially between subcortical and cortical regions but was generally positive at the group level. In comparison to the other networks, the SC-FC coupling in the ventral attention and somatomotor areas was generally higher (0.29 ± 0.40 and 0.23 ± 0.20, respectively). In contrast, the coupling in the subcortical and dorsal attention areas was significantly weaker (-0.06 ± 0.16 and -0.12 ± 0.23, respectively) (**Figure 2**).

### 3.3 Association of prediabetes and T2DM with regional SC-FC coupling

T2DM was significantly associated with weaker regional SC-FC coupling in the limbic areas (β = -0.110; 95% CI: -0.196 to -0.024; *P* = 0.012) and higher coupling in the default mode areas (β = 0.093; 95% CI: 0.028 to 0.158; *P* = 0.005) after full adjustment. The regression coefficients for the relationship between prediabetes and coupling in the limbic and default mode areas were roughly one-third to two-thirds of the coefficient for T2DM. There were no correlations between T2DM or prediabetes and SC-FC coupling in the remaining domains (**Table 2**).

**Table 2:** Association of prediabetes and T2DM with SC-FC coupling after multivariate adjustment.

Brain network	Prediabetes, β (95% CI)	*P*	T2DM, β (95% CI)	*P*
Visual				
Model 1	0.019 (-0.024, 0.062)	0.379	-0.021 (-0.059, 0.016)	0.262
Model 2	0.005 (-0.040, 0.051)	0.819	-0.029 (-0.071, 0.013)	0.178
Somatomotor				
Model 1	-0.018 (-0.073, 0.037)	0.521	-0.022 (-0.067, 0.023)	0.331
Model 2	-0.018 (-0.075, 0.040)	0.547	-0.020 (-0.071, 0.030)	0.425
Dorsal Attention				
Model 1	0.009 (-0.053, 0.072)	0.772	0.045 (-0.010, 0.099)	0.110
Model 2	0.001 (-0.066, 0.067)	0.982	0.048 (-0.014, 0.109)	0.129
Ventral Attention				
Model 1	-0.008 (-0.117, 0.102)	0.893	0.022 (-0.068, 0.112)	0.630
Model 2	-0.027 (-0.143, 0.088)	0.640	0.027 (-0.073, 0.127)	0.593
Limbic				
Model 1	-0.040 (-0.128, 0.049)	0.380	**-0.106 (-0.183, -0.029)**	**0.007**
Model 2	-0.038 (-0.131, 0.055)	0.419	**-0.110 (-0.196, -0.024)**	**0.012**
Frontoparietal				
Model 1	-0.004 (-0.080, 0.072)	0.924	0.001 (-0.064, 0.067)	0.974
Model 2	0.003 (-0.077, 0.084)	0.940	0.010 (-0.063, 0.083)	0.780
Default				
Model 1	0.046 (-0.025, 0.117)	0.205	**0.087 (0.029, 0.146)**	**0.003**
Model 2	0.030 (-0.045, 0.105)	0.426	**0.093 (0.028, 0.158)**	**0.005**
Subcortical				
Model 1	0.007 (-0.033, 0.047)	0.740	-0.001 (-0.036, 0.034)	0.949
Model 2	0.003 (-0.039, 0.045)	0.874	-0.010 (-0.049, 0.029)	0.607

Normal glucose metabolism as a reference. Mean differences in networks of patients with prediabetes or T2DM compared with NGM are expressed as regression coefficients and 95% CIs. Boldface type indicates *P* < 0.05.

### 3.4 Relationship between diabetes-related measures and regional SC-FC coupling

After full adjustment, fasting glucose and HbA_1c_ were associated with weaker regional SC-FC coupling in the limbic areas (β = -0.095; 95% CI: -0.189 to -0.001; *P* = 0.049; and β = -0.106; 95% CI: -0.201 to -0.011; *P* = 0.029). Fasting insulin was associated with higher coupling in the limbic areas (β = 0.115; 95% CI: 0.021 to 0.209; *P* = 0.017), and fasting insulin contributed the most to the change of coupling. In addition, HbA_1c_ was associated with higher coupling in the dorsal attention areas (β = 0.113; 95% CI: 0.018 to 0.209; *P* = 0.020) (**Table 3**).

**Table 3:** Multivariable-adjusted associations between diabetes-related measures, structural-functional connectivity coupling, and MoCA score.

	Fasting glucose	*P*	HbA_1c_	*P*	Fasting insulin	*P*
Visual						
Model 1	-0.017 (-0.108, 0.074)	0.710	-0.036 (-0.127, 0.054)	0.429	0.024 (-0.065, 0.113)	0.593
Model 2	-0.032 (-0.128, 0.063)	0.504	-0.046 (-0.142, 0.049)	0.343	0.002 (-0.093, 0.097)	0.965
Somatomotor						
Model 1	0.010 (-0.080, 0.100)	0.827	-0.009 (-0.099, 0.081)	0.841	0.008 (-0.080, 0.096)	0.861
Model 2	0.015 (-0.079, 0.109)	0.759	-0.007 (-0.102, 0.088)	0.885	0.007 (-0.087, 0.101)	0.877
Dorsal Attention						
Model 1	0.063 (-0.028, 0.154)	0.173	**0.107 (0.017, 0.198)**	**0.020**	0.038 (-0.052, 0.127)	0.408
Model 2	0.061 (-0.035, 0.156)	0.213	**0.113 (0.018, 0.209)**	**0.020**	0.023 (-0.072, 0.119)	0.634
Ventral Attention						
Model 1	0.020 (-0.071, 0.110)	0.668	0.015 (-0.075, 0.105)	0.743	0.030 (-0.059, 0.119)	0.506
Model 2	0.009 (-0.086, 0.103)	0.852	0.007 (-0.088, 0.103)	0.877	0.027 (-0.067, 0.122)	0.568
Limbic						
Model 1	**-0.093 (-0.183, -0.002)**	**0.044**	**-0.112 (-0.202, -0.022)**	**0.015**	**0.106 (0.017, 0.194)**	**0.019**
Model 2	**-0.095 (-0.189, -0.001)**	**0.049**	**-0.106 (-0.201, -0.011)**	**0.029**	**0.115 (0.021, 0.209)**	**0.017**
Frontoparietal						
Model 1	0.002 (-0.089, 0.092)	0.971	0.020 (-0.070, 0.111)	0.656	0.054 (-0.034, 0.143)	0.229
Model 2	0.021 (-0.074, 0.115)	0.665	0.044 (-0.051, 0.139)	0.365	0.070 (-0.024, 0.164)	0.146
Default						
Model 1	0.028 (-0.062, 0.119)	0.538	0.043 (-0.047, 0.133)	0.347	0.026 (-0.062, 0.114)	0.564
Model 2	0.019 (-0.076, 0.113)	0.700	0.044 (-0.050, 0.139)	0.358	0.022 (-0.072, 0.116)	0.643
Subcortical						
Model 1	0.048 (-0.042, 0.137)	0.296	0.057 (-0.031, 0.146)	0.204	-0.056 (-0.143, 0.031)	0.208
Model 2	0.029 (-0.064, 0.122)	0.546	0.048 (-0.045, 0.142)	0.313	-0.077 (-0.169, 0.016)	0.104
MoCA						
Model 1	**-0.149 (-0.225, -0.073)**	**<0.001**	**-0.146 (-0.222, -0.070)**	**<0.001**	0.038 (-0.037, 0.114)	0.318
Model 2	**-0.171 (-0.250, -0.092)**	**<0.001**	**-0.185 (-0.264, -0.106)**	**<0.001**	0.056 (-0.024, 0.137)	0.167

Boldface type indicates *P* < 0.05.

### 3.5 Mediating role of cerebral regional SC-FC coupling in the relationship between diabetes-related measures and MoCA score

**Figure 3:**
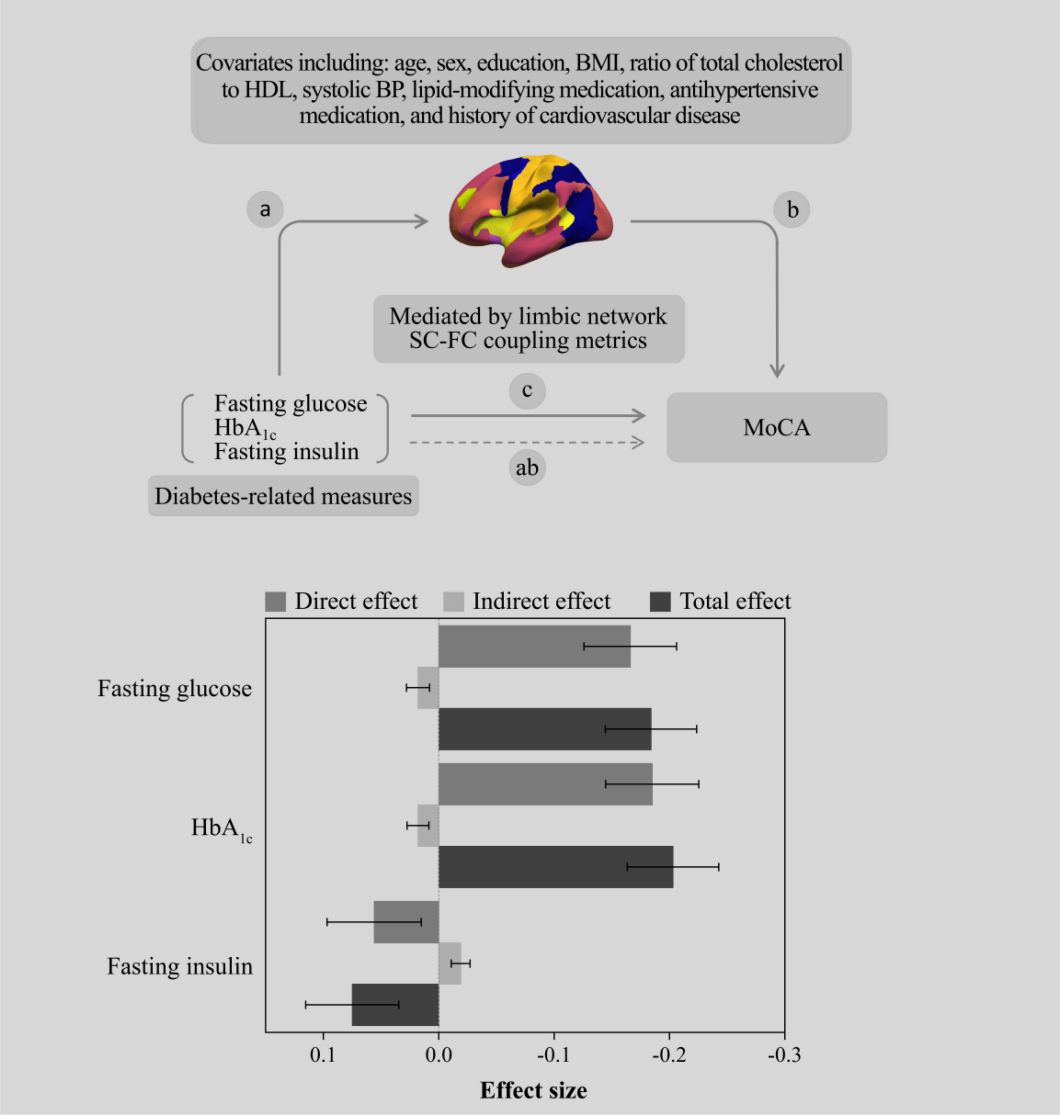
The SC-FC coupling metrics of the limbic network suppress the association between diabetes-related measures and the MoCA score.

Considering the association of “diabetes-related measures—regional SC-FC coupling” and “regional SC-FC coupling—MoCA score” (**Supplementary Table S2**), we estimated the single mediating effect of SC-FC coupling levels in limbic areas on the association of “diabetes-related measures—MOCA score”. The study revealed that the suppressing effect of SC-FC coupling in limbic areas on the relationship between fasting insulin and MoCA score was strongest (β_indirect effect_ = -0.019, 95% CI: -0.034, -0.001), with a suppressing effect of 25.3%. In addition, significant indirect effects could also be found in fasting glucose (β_indirect effect_ = 0.018, 95% CI: 0.001, 0.040) and HbA_1c_ (β_indirect effect_ = 0.018, 95% CI: 0.002, 0.039), corresponding to a suppressing effect of 9.8% and 8.9%, respectively (**Figure 3**, **Supplementary Table S3**).

### 3.6 Additional analyses

Tractography algorithms are recognized to underestimate connections between the two hemispheres. When we consider the unilateral brain separately, we found differences in SC-FC coupling regions only in the dominant brain (left brain); T2DM was significantly associated with higher coupling in the default mode areas (β = 0.150; 95% CI: 0.073 to 0.227; *P <*0.001) compared with NGM (**Supplementary Table S4**). No associations between prediabetes and T2DM were found with regional SC-FC coupling in the nondominant brain (right brain) (**Supplementary Table S5**). Finally, we found similar results when we used diabetic status instead of diabetes-related measures to assess whether SC-FC coupling mediates the relationship between diabetic status and cognition (**Supplementary Table S6**).

## 4 Discussion

This population-based study suggests that there are differences in SC-FC coupling between different brain networks. Thus, our results support the notion that information processing in the cerebral cortex involves the existence of interactions and hierarchies between distributed regions (19, 22). In contrast to what we found, recent HCP research in young populations has shown strongest SC-FC coupling in the subcortical and visual region (23). This may be due to racial and age differences in the sample population, as the HCP study population was a young cohort, and there is evidence that early visual areas are strongly functionally coupled to each other, with little correlation to areas outside of the visual cortex (19). In addition, the HCP quantifies SC-FC coupling by calculating Spearman’s correlations between each region and other regions in the same network, whereas we are extracting the vector of non-zero structural connectivity values first and then the corresponding vector of functional connectivity for Spearman correlation. Future studies will need to include different age groups and multi-center sample populations to test this hypothesis.

Compared with NGM, T2DM patients had lower coupling of the limbic network and higher coupling of the default mode network. When we divided the brain into left and right hemispheres for separate analysis, we found higher SC-FC coupling of the default mode network in T2DM patients than in NGM only on the dominant cerebral side, which we hypothesized to be due to the presence of interhemispheric lateralization in T2DM-associated brain injuries (24) or to underestimation of cross-hemispheric connectivity by neural tractography algorithms (25). In this study, a general linear model was used to validate brain coupling progression from NGM to prediabetes to T2DM. In fact, the increase in prediabetic brain coupling abnormalities is about one-third to two-thirds that of T2DM. These findings suggest that changes in the brains of people with prediabetes have already occurred prior to the clinical diagnosis of T2DM. Therefore, prediabetes treatment should be considered as a potential intervention target to prevent complications of T2DM. To my knowledge, this is the first research in the realm of diabetes to discuss SC-FC coupling. Therefore, this paper extends the previous results by describing SC-FC coupling in the network region.

Our study observed significant correlations of diabetes-related measures with limbic network SC-FC coupling and MoCA. For example, for every 1 unit increase in HbA_1c_, MoCA decreased by 0.146 (95% CI: 0.070, 0.222), and the SC-FC coupling level of the limbic network decreased by 0.106 (95% CI: 0.011, 0.201). These associations suggest that diabetes-related measures may affect the normal SC-FC coupling and cognitive function. Although this is the first article to explore the correlation between diabetes-related measures and cerebral coupling, a large body of research suggests that abnormal structural and functional connectivity may be caused by hyperglycemia and insulin resistance (26, 27). Therefore, we speculate that the SC-FC coupling will also change correspondingly. Interestingly, this study found that only limbic network SC-FC coupling was linked with cognition. It is well known that limbic network areas such as the prefrontal cortex, hippocampus, and basolateral amygdala are closely related to learning processes and memory (28), which is consistent with previous findings of abnormal functional and structural connections of limbic regions in T2DM (12, 29). We further estimated whether the limbic network coupling could mediate the association of “diabetes-related measures—MoCA.” However, we found that the indirect and direct effects of this mediation model were in opposite directions, suggesting that the mediating variable (SC-FC coupling of the limbic regions) suppressed the association between T2DM and cognitive decline. In other words, decreased SC-FC coupling of the limbic regions may be a potential compensatory mechanism for T2DM associated with cognitive dysfunction. In additional analysis, we found that the limbic network coupling of T2DM with normal cognition (MoCA ≥26) was weaker than that of T2DM with cognitive impairment (MoCA < 26), with values of 0.03 ± 0.33 and 0.08 ± 0.34, respectively (**Supplementary Figure S2**). Our hypothesis posits that the limbic network region underwent rearrangement in order to compensate for impaired cognitive function, resulting in changes in the link between structural and functional connectivity. Neuroplasticity mechanisms can help explain the observed potential compensatory phenomena described above. Neuroplasticity refers to the capacity of the nervous system to adapt and restructure its organization and functioning in response to stimuli originating from within or outside the body (30, 31). Hyperglycemia and hyperinsulinemia can affect neuroplasticity pathways (32, 33), thus affecting learning and memory (34, 35). Another study suggests that variations in memory processes may be caused by decreased synaptic communication resulting from changes in the dendritic arborization and the number of dendritic spines in limbic areas such as the prefrontal cortex and hippocampus (28).

The strength of this study is that we conducted a comprehensive analysis of brain SC-FC coupling in patients with T2DM and prediabetes using multimodal MRI, which for the first time provides data on the association between glucose metabolism status and SC-FC coupling. Second, our mediation model allows us to address the question of whether diabetes-related measures are independently associated with observed compensation for cognitive function, extending our current understanding of the pathogenesis of T2DM. There are some limitations to this study. We assessed prediabetes and normal glucose metabolic status by FPG and HbA_1c_ markers, which may have led to the misclassification of participants with impaired glucose tolerance status. Second, it is hard to investigate casual associations because the study was designed in a cross-sectional way. Therefore, future longitudinal research is required to determine whether hyperglycemia precedes the development of reported abnormalities in SC-FC coupling.

## 5 Conclusion

We observed a significant relationship between T2DM, diabetes-related measures, and cerebral SC-FC coupling. In addition, we provide preliminary evidence that the level of limbic network coupling may have a suppressing effect on the relationship between diabetic-related measures and cognitive function. These findings support the concept that the relationship between limbic network functional and structural connectivity reorganizes to compensate for impaired cognitive function and that this phenomenon may begin with prediabetes. These findings will enhance comprehension of cognitive mechanisms in T2DM and prediabetes and aid in the development of therapies to prevent neurological illnesses associated with diabetes.

## Data Availability

The raw data supporting the conclusions of this article will be made available by the authors, without undue reservation.
