# Supplementary material for: Potential compensatory mechanism for cognitive impairment in type 2 diabetes and prediabetes: altered structure-function coupling
Source: Front Endocrinol (Lausanne). 2025 Mar 17;16:1491377. doi: 10.3389/fendo.2025.1491377 (PMC11955491; doi:10.3389/fendo.2025.1491377)
Supplement: Supplementary file 1 [file DataSheet1.docx]

**Supplementary Materials**

**Supplementary Figure 1.** Flowchart of the MRI study population


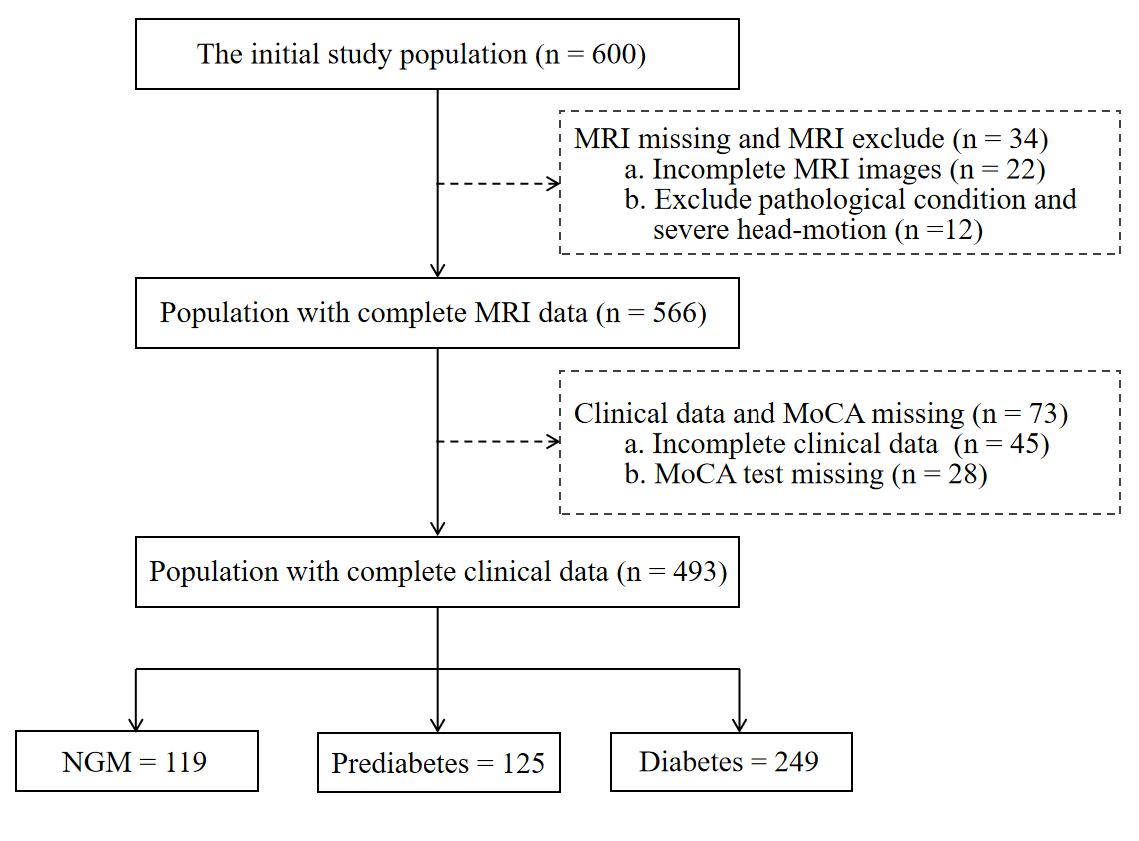


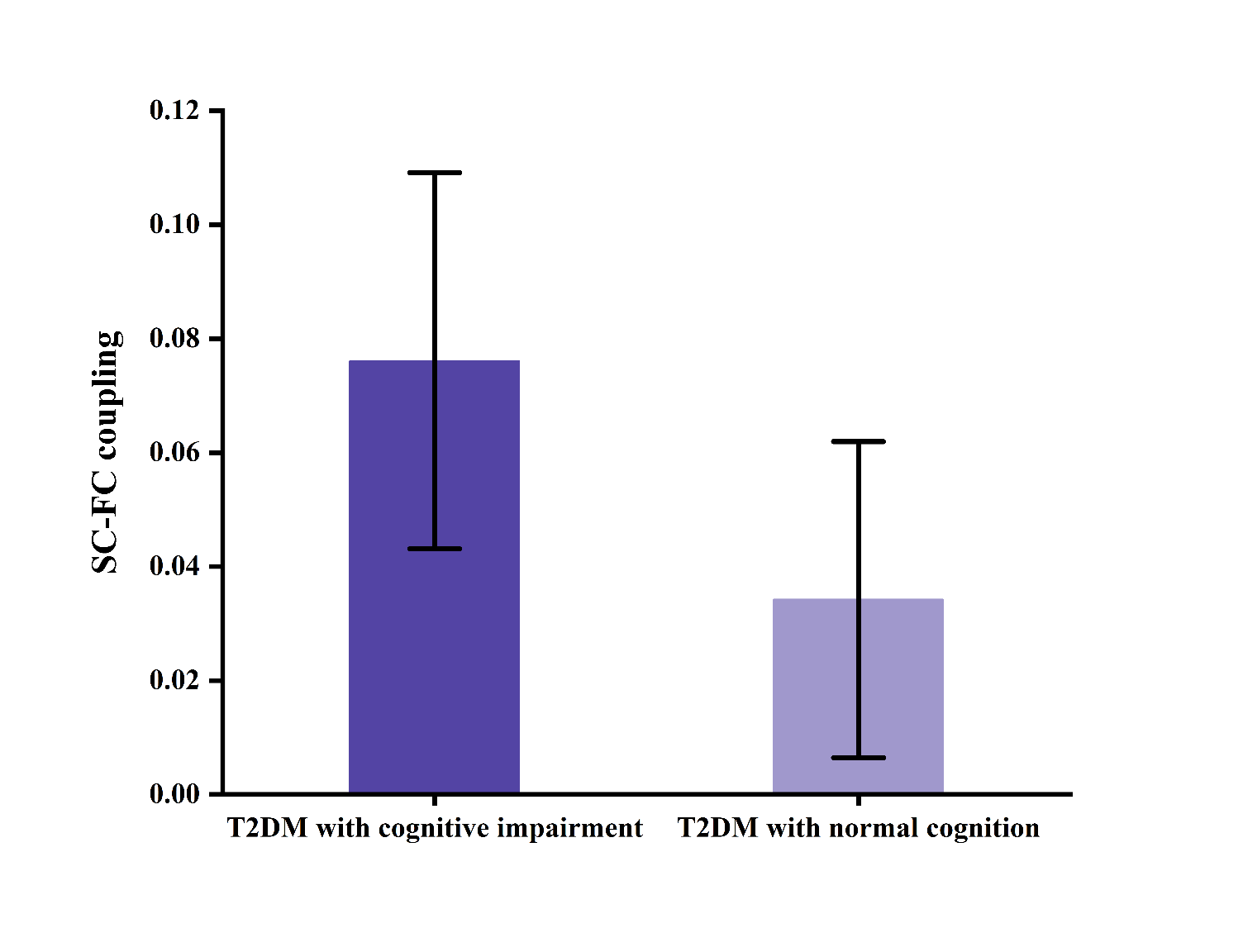


**Supplementary Figure 2.** SC-FC coupling level in limbic network areas for T2DM with cognitive impairment and T2DM with normal cognition.

**Supplementary Table 1.** MRI acquisition protocols

| Sequence | TR/TE (ms) | Slice thickness (mm)/gap | FOV (mm^2^) | Voxel size (mm^3^) | Acquisition time (min) |
| --- | --- | --- | --- | --- | --- |
| T1WI | 2530/2.98 | 1/0 | 256 × 256 | 1 × 1 × 1 | 5:58 |
| fMRI | 500/30 | 3.5/0.5 | 224 × 256 | 3.5 × 3.5 × 4 | 8:07 |
| dMRI | 4200/72 | 2/1 | 220 × 220 | 2 × 2 × 2 | 7:31 |

* The diffusion imaging data includes 11 b-values (b = 300, 350, 650, 950, 1000, 1350, 1650, 1700, 2000, 2700, and 3000 s/mm^2^) along the direction of 99 diffusion gradients, with two b = 0 s/mm^2^ images and a phase coded image in opposite directions.

**Supplementary Table 2.** Multivariable-adjusted associations between structural-functional connectivity coupling and MoCA score

|  | MoCA | *P* |
| --- | --- | --- |
| Visual |  |  |
| Model 1 | -0.045 (-0.120, 0.031) | 0.245 |
| Model 2 | -0.042 (-0.118, 0.033) | 0.272 |
| Somatomotor |  |  |
| Model 1 | -0.008 (-0.084, 0.068) | 0.839 |
| Model 2 | -0.003 (-0.079, 0.074) | 0.942 |
| Dorsal Attention |  |  |
| Model 1 | -0.013 (-0.088, 0.063) | 0.736 |
| Model 2 | -0.015 (-0.091, 0.060) | 0.687 |
| Ventral Attention |  |  |
| Model 1 | -0.005 (-0.081, 0.071) | 0.900 |
| Model 2 | -0.003 (-0.079, 0.074) | 0.947 |
| Limbic |  |  |
| Model 1 | **-0.162 (-0.236, -0.088)** | **<0.001** |
| Model 2 | **-0.154 (-0.229, -0.079)** | **<0.001** |
| Frontoparietal |  |  |
| Model 1 | 0.062 (-0.014, 0.137) | 0.111 |
| Model 2 | 0.064 (-0.012, 0.140) | 0.096 |
| Default |  |  |
| Model 1 | 0.002 (-0.074, 0.078) | 0.965 |
| Model 2 | 0.006 (-0.070, 0.082) | 0.881 |
| Subcortical |  |  |
| Model 1 | -0.029 (-0.106, 0.048) | 0.458 |
| Model 2 | -0.028 (-0.105, 0.050) | 0.482 |

Boldface type indicates *P* < 0.05.

**Supplementary Table 3.** Estimated total effect, direct effects, and indirect effects mediated by structural-functional connectivity coupling for the association between diabetes-related measures and MoCA after full adjustment

| Independent variable x | Dependent variable y | Mediator M | Total effect (95% CI) | Direct effect (95% CI) | Indirect effect (95% CI) |
| --- | --- | --- | --- | --- | --- |
| Fasting glucose | MoCA | SC-FC coupling of the limbic network | -0.166 (-0.245, -0.087) | -0.184 (-0.261, -0.106) | 0.018 (0.001, 0.040) |
| HbA_1c_ |  |  | -0.185 (-0.264, -0.106) | -0.203 (-0.281, -0.125) | 0.018 (0.002, 0.039) |
| Fasting insulin |  |  | 0.056 (-0.024, 0.137) | 0.075 (-0.004, 0.154) | -0.019 (-0.034, -0.001) |

**Supplementary Table 4.** Association of prediabetes and T2DM with SC-FC coupling of dominant cerebral hemisphere after multivariate adjustment

| Brain network | Prediabetes, β (95% CI) | *P* | T2DM, β (95% CI) | *P* |
| --- | --- | --- | --- | --- |
| Visual |  |  |  |  |
| Model 1 | 0.044 (-0.022, 0.110) | 0.191 | -0.044 (-0.100, 0.011) | 0.118 |
| Model 2 | 0.041 (-0.028, 0.111) | 0.245 | -0.055 (-0.117, 0.008) | 0.087 |
| Somatomotor |  |  |  |  |
| Model 1 | 0.012 (-0.069, 0.094) | 0.763 | -0.064 (-0.138, 0.010) | 0.088 |
| Model 2 | 0.014 (-0.072, 0.100) | 0.750 | -0.070 (-0.152, 0.012) | 0.095 |
| Dorsal Attention |  |  |  |  |
| Model 1 | -0.018 (-0.091, 0.055) | 0.631 | 0.030 (-0.037, 0.097) | 0.372 |
| Model 2 | -0.027 (-0.104, 0.051) | 0.500 | 0.032 (-0.044, 0.107) | 0.409 |
| Ventral Attention |  |  |  |  |
| Model 1 | -0.029 (-0.178, 0.120) | 0.698 | 0.037 (-0.087, 0.160) | 0.560 |
| Model 2 | -0.059 (-0.217, 0.098) | 0.458 | 0.007 (-0.130, 0.143) | 0.925 |
| Limbic |  |  |  |  |
| Model 1 | -0.094 (-0.215, 0.026) | 0.125 | -0.104 (-0.212, 0.005) | 0.061 |
| Model 2 | -0.070 (-0.196, 0.056) | 0.276 | -0.103 (-0.224, 0.018) | 0.094 |
| Frontoparietal |  |  |  |  |
| Model 1 | 0.016 (-0.151, 0.182) | 0.853 | 0.075 (-0.071, 0.222) | 0.313 |
| Model 2 | -0.015 (-0.191, 0.161) | 0.865 | 0.056 (-0.107, 0.218) | 0.503 |
| Default |  |  |  |  |
| Model 1 | 0.074 (-0.014, 0.162) | 0.100 | **0.126 (0.056, 0.195)** | **<0.001** |
| Model 2 | 0.062 (-0.028, 0.153) | 0.177 | **0.150 (0.073, 0.227)** | **<0.001** |
| Subcortical |  |  |  |  |
| Model 1 | -0.003 (-0.054, 0.048) | 0.912 | 0.001 (-0.044, 0.046) | 0.977 |
| Model 2 | -0.001 (-0.054, 0.052) | 0.970 | -0.004 (-0.054, 0.046) | 0.875 |

Normal glucose metabolism as a reference. Mean differences in networks of patients with prediabetes or T2DM compared with NGM are expressed as regression coefficients and 95% CIs. Boldface type indicates *P* < 0.05.

**Supplementary Table 5.** Association of prediabetes and T2DM with SC-FC coupling of non-dominant cerebral hemisphere after multivariate adjustment

| Brain network | Prediabetes, β (95% CI) | *P* | T2DM, β (95% CI) | *P* |
| --- | --- | --- | --- | --- |
| Visual |  |  |  |  |
| Model 1 | 0.025 (-0.031, 0.082) | 0.379 | 0.007 (-0.042, 0.056) | 0.788 |
| Model 2 | 0.001 (-0.059, 0.060) | 0.985 | -0.004 (-0.059, 0.051) | 0.877 |
| Somatomotor |  |  |  |  |
| Model 1 | 0.007 (-0.084, 0.099) | 0.875 | 0.030 (-0.045, 0.106) | 0.432 |
| Model 2 | -0.002 (-0.096, 0.093) | 0.975 | 0.033 (-0.052, 0.117) | 0.449 |
| Dorsal Attention |  |  |  |  |
| Model 1 | 0.060 (-0.045, 0.164) | 0.261 | 0.067 (-0.018, 0.152) | 0.121 |
| Model 2 | 0.055 (-0.056, 0.166) | 0.329 | 0.085 (-0.010, 0.180) | 0.079 |
| Ventral Attention |  |  |  |  |
| Model 1 | 0.042 (-0.053, 0.137) | 0.387 | 0.038 (-0.042, 0.118) | 0.352 |
| Model 2 | 0.058 (-0.043, 0.159) | 0.260 | 0.001 (-0.088, 0.088) | 0.999 |
| Limbic |  |  |  |  |
| Model 1 | -0.120 (-0.290, 0.050) | 0.167 | -0.079 (-0.230, 0.073) | 0.308 |
| Model 2 | -0.131 (-0.311, 0.048) | 0.150 | -0.115 (-0.282, 0.053) | 0.179 |
| Frontoparietal |  |  |  |  |
| Model 1 | -0.026 (-0.116, 0.063) | 0.562 | -0.059 (-0.134, 0.016) | 0.123 |
| Model 2 | -0.005 (-0.100, 0.089) | 0.915 | -0.036 (-0.119, 0.048) | 0.398 |
| Default |  |  |  |  |
| Model 1 | -0.055 (-0.200, 0.090) | 0.457 | -0.011 (-0.138, 0.116) | 0.862 |
| Model 2 | -0.096 (-0.250, 0.058) | 0.220 | -0.003 (-0.145, 0.139) | 0.968 |
| Subcortical |  |  |  |  |
| Model 1 | 0.015 (-0.037, 0.067) | 0.578 | -0.011 (-0.056, 0.035) | 0.645 |
| Model 2 | 0.007 (-0.048, 0.061) | 0.816 | -0.027 (-0.078, 0.023) | 0.286 |

Normal glucose metabolism as a reference. Mean differences in networks of patients with prediabetes or T2DM compared with NGM are expressed as regression coefficients and 95% CIs. Boldface type indicates *P* < 0.05.

**Supplementary Table 6.** Estimated direct effects, indirect effects of structural-functional connectivity coupling in model 2 for the association between diabetes status and MoCA

| Structural-functional connectivity coupling | Direct effect (95% CI) | Indirect effect (95% CI) |
| --- | --- | --- |
| Visual | -0.087 (-0.190, 0.017) | 0.005 (-0.003, 0.019) |
| Somatomotor | -0.081 (-0.185, 0.022) | 0.001 (-0.004, 0.006) |
| Dorsal Attention | -0.080 (-0.184, 0.024) | -0.001 (-0.012, 0.008) |
| Ventral Attention | -0.081 (-0.185, 0.022) | -0.001 (-0.006, 0.005) |
| Limbic | **-0.110 (-0.213, -0.008)** | **0.029 (0.007, 0.054)** |
| Frontoparietal | -0.083 (-0.186, 0.020) | 0.002 (-0.008, 0.012) |
| Default | -0.084 (-0.188, 0.021) | 0.002 (-0.012, 0.018) |
| Subcortical | -0.083 (-0.187, 0.021) | 0.002 (-0.004, 0.009) |

Boldface type indicates *P* < 0.05.
